# Supplementary material for: Octopamine Rescues Endurance and Climbing Speed in Drosophila Clkout Mutants with Circadian Rhythm Disruption
Source: Cells. 2023 Oct 25;12(21):2515. doi: 10.3390/cells12212515 (PMC10648926; doi:10.3390/cells12212515)
Supplement: Supplementary file 1 [file cells-12-02515-s001.zip › Supplementary Figures.pdf]

Supplementary Figures

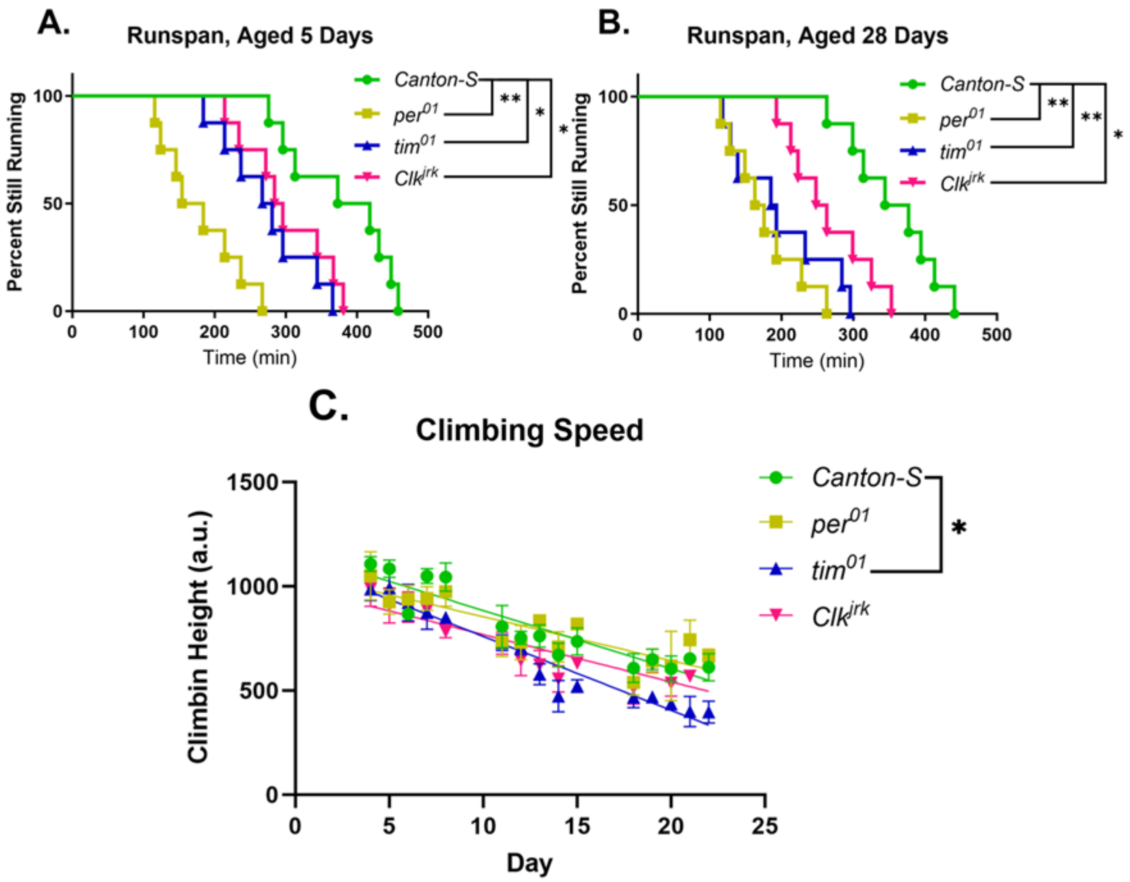

Supplemental Figure S1: Mobility associated phenotypes of circadian mutants in the *Canton-S* background *tim^{01}*, *per^{01}*, and *Clk^{jrk}*, show reduced endurance when compared to *Canton-S* control flies early in life at five days of age (A) and later in life at 28 days of age, n=8 vials of 20 flies each, (log-rank analysis) (B). *Clk^{jrk}* and *per^{01}* mutants show no significant changes in climbing speed compared to *Canton-S* while *tim^{01}* mutants showed reduced climbing speed in the second and third week of life, n=5 vials of 20 flies each, 2-way ANOVA and trendline represents linear regression (C). *$p<0.05$, **$p<0.001$.

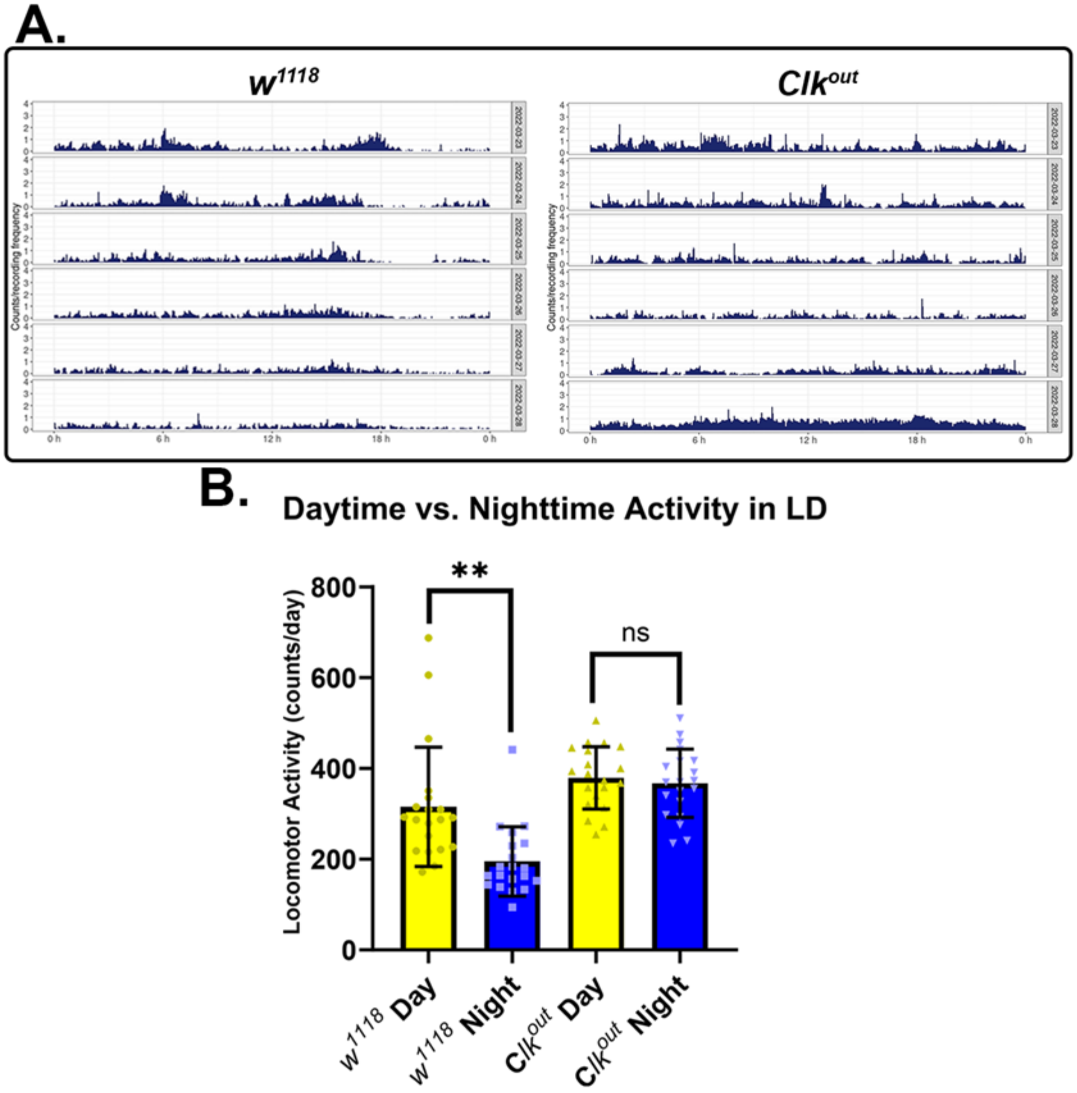

Supplemental Figure S2: *Clk^out* mutants have a disrupted circadian rhythm. The *Drosophila* activity monitor was used to assess the activity rhythm of *Clk^out* flies and controls. *Clk^out* flies had no discernable rhythm on actograms whereas the control flies maintained a rhythm for some days of the assessment, n=16 (A). *Clk^out* flies had no significant change in activity with day versus night conditions whereas controls had a significant reduction in activity within the night-time hours, n=16 (B). n=20, t-test, **$p<0.01$. Actograms were generated using the ShinyR DAM analysis app.

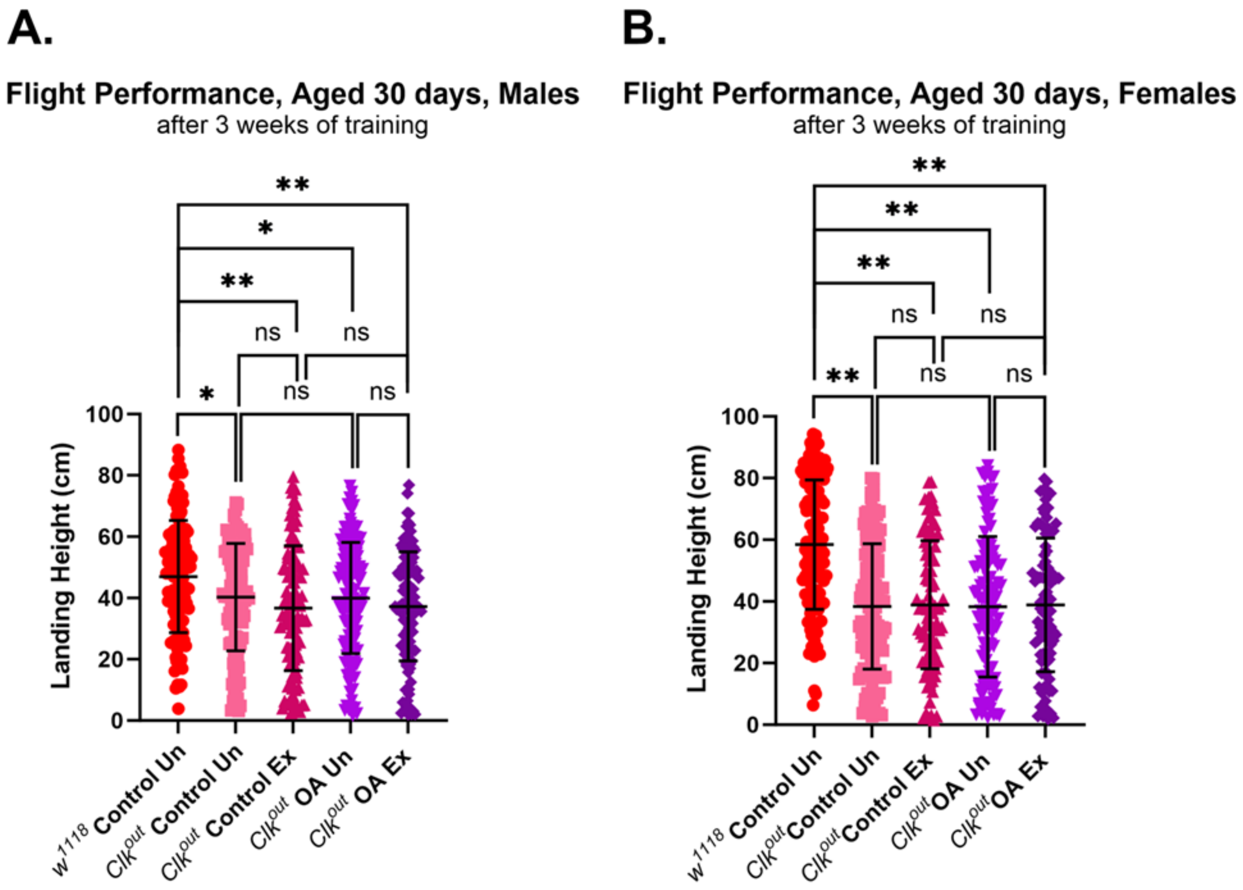

Supplemental Figure S3: Octopamine feeding does not rescue flight performance in *Clk^out^* mutants. Flight performance was assessed in male and female *Clk^out^* mutants fed OA for three weeks. Neither males nor females showed any significant improvements in flight performance with this treatment. Endurance exercise also did not impact flight performance in either sex with or without OA feeding. All mutants had a significantly worse flight performance when compared to unexercised w^{1118} controls. n=8 vials of 20 flies each, (t-test). *p<0.05, **p<0.001.

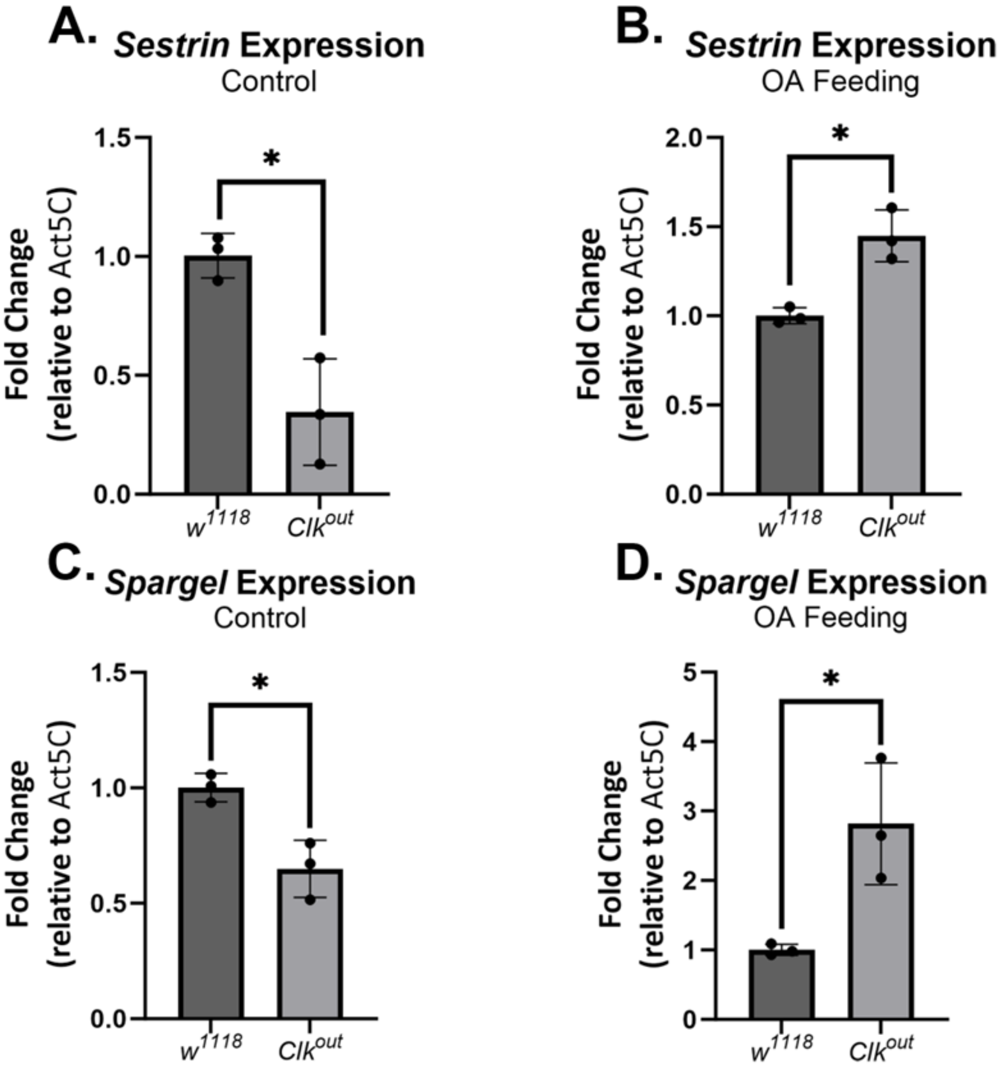

Supplemental Figure S4: *Clk^{out}* mutants have low expression of exercise response genes and Octopamine feeding increases their expression. qRT-PCR was used with the *delta delta Ct* method to compare gene expression of key exercise response genes between *Clk*^{out} mutants and *w*^{1118} controls. Mutants had reduced expression of both *sestrin* and *spargel* (A, C). After five days of OA feeding the expression of *sestrin* and *spargel* in the mutants increased significantly (B, D). n=3 biological replicates, t-test, *$p<0.05$.

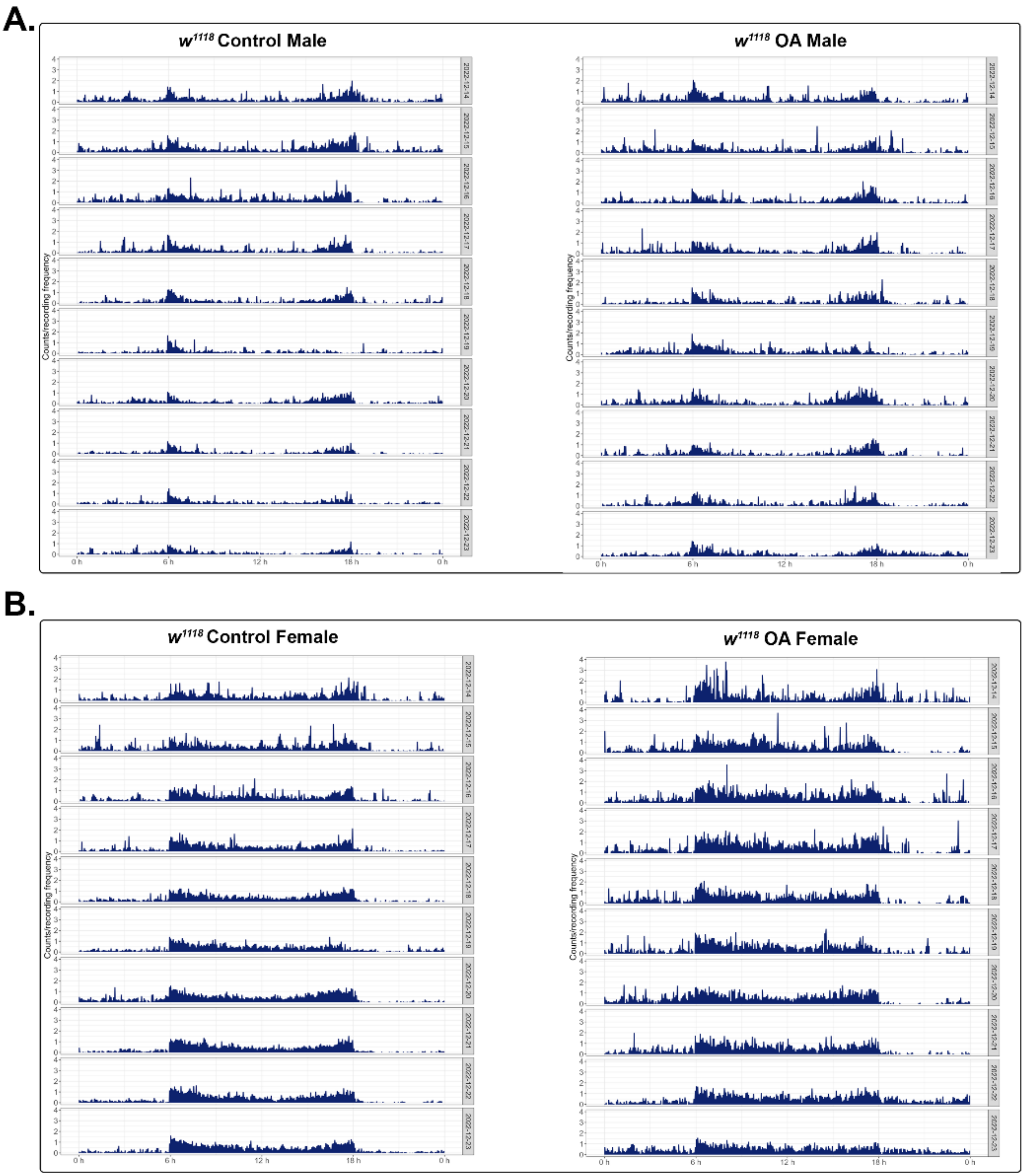
A.
w^{1118} Control Male
w^{1118} OA Male
B.
w^{1118} Control Female
w^{1118} OA Female
Counts/recording frequency
0 h
6 h
12 h
18 h
0 h
2022-12-14
2022-12-15
2022-12-16
2022-12-17
2022-12-18
2022-12-19
2022-12-20
2022-12-21
2022-12-22
2022-12-23

C.

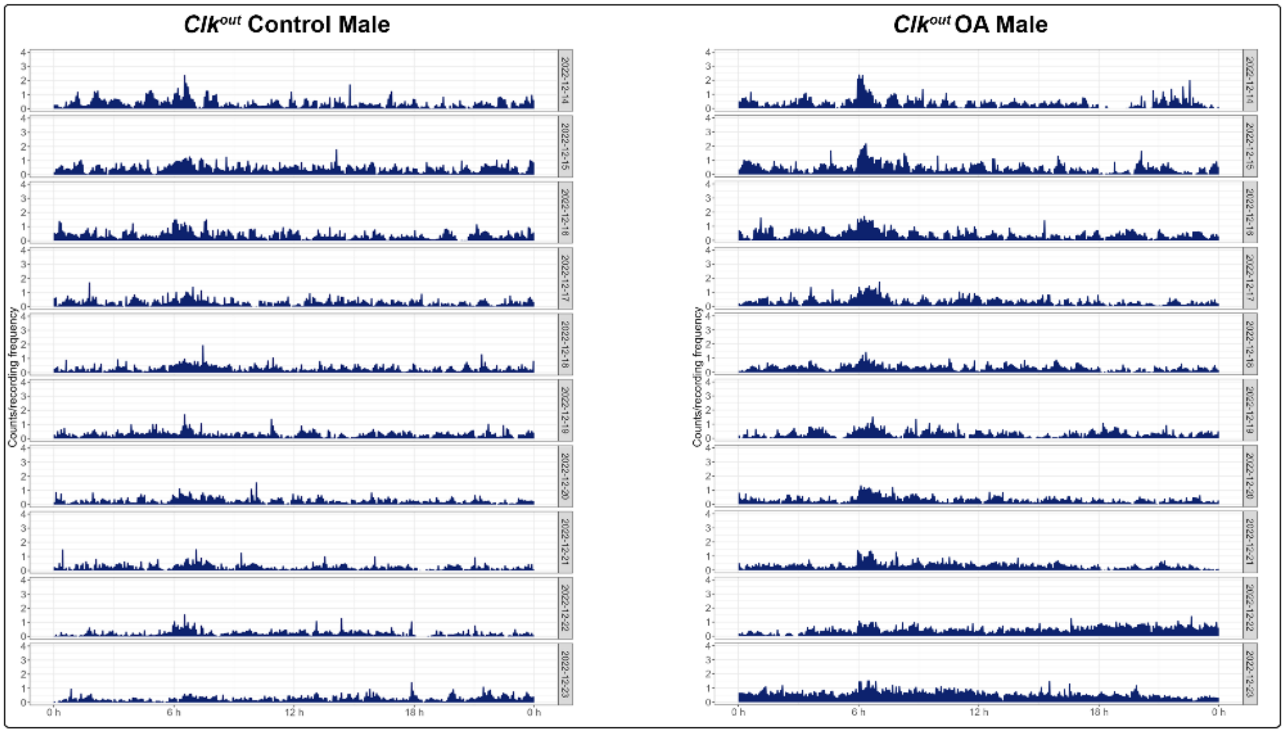

*Clk*ᵒᵘᵗ Control Male
*Clk*ᵒᵘᵗ OA Male
Counts/recording frequency
0 h
6 h
12 h
18 h
0 h

D.

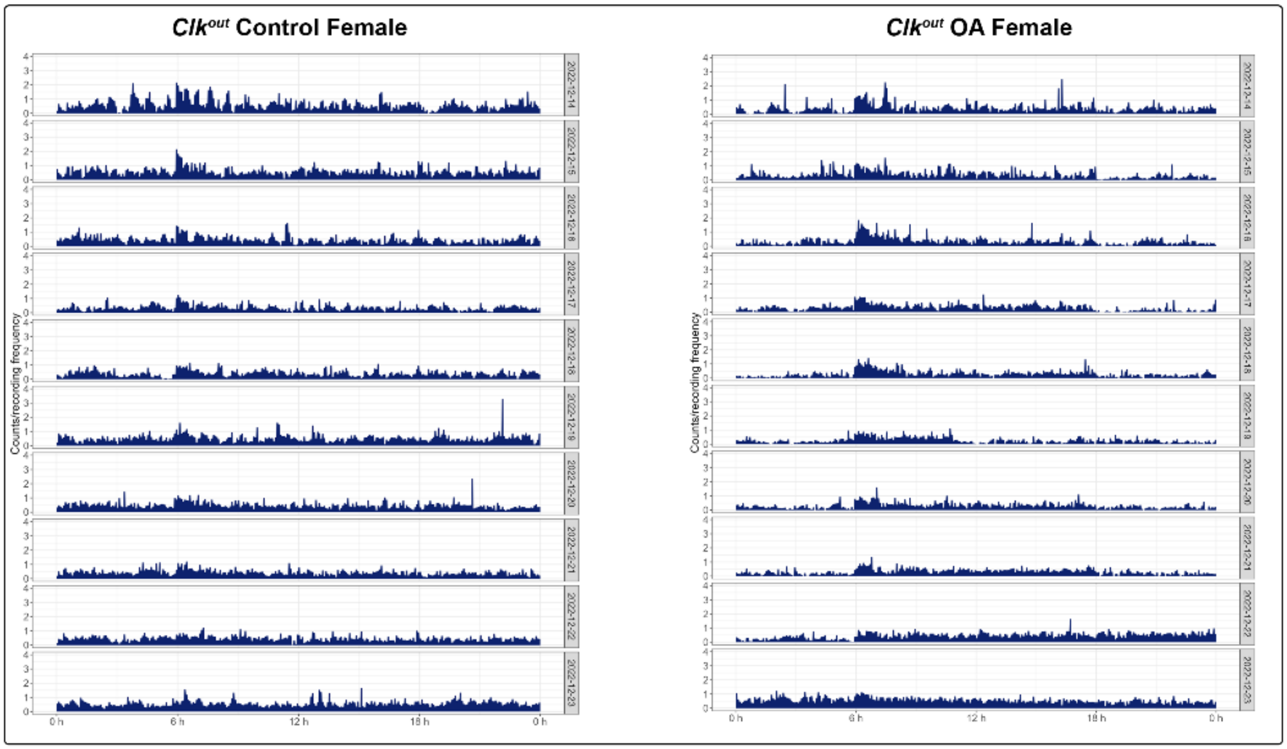

*Clk*ᵒᵘᵗ Control Female
*Clk*ᵒᵘᵗ OA Female
Counts/recording frequency
0 h
6 h
12 h
18 h
0 h

Supplemental Figure S5: Octopamine feeding does not impact activity profiles of control or mutant flies. *w^{1118}* males display a regular activity rhythm which is preserved without entraining light signals and their activity profile does not change with OA treatment (A). *w^{1118}* females also display a similar rhythm as the males and while they do show some increased activity with OA feeding, their activity profile remains mostly unchanged (B). *Clk^{out}* males and females do not have a discernable activity rhythm without light entrainment signals and do not show any changes to their activity profiles with OA treatment (C, D). The actograms show data collected over the course of 10 days; light entrainment was provided for the first three days (LD) while the last seven days were assessed in complete darkness (DD). Actograms were generated using the ShinyR DAM analysis app. Time 0h represents 12 am.
